# Charting Tomorrow’s Healthcare: A Traditional Literature Review for an Artificial Intelligence-Driven Future

**DOI:** 10.7759/cureus.58032

**Published:** 2024-04-11

**Authors:** Brody M Fogleman, Matthew Goldman, Alexander B Holland, Garrett Dyess, Aashay Patel

**Affiliations:** 1 Internal Medicine, Edward Via College of Osteopathic Medicine - Carolinas, Spartanburg, USA; 2 Neurological Surgery, Houston Methodist Hospital, Houston, USA; 3 General Surgery, Edward Via College of Osteopathic Medicine - Carolinas, Spartanburg, USA; 4 Medicine, University of South Alabama College of Medicine, Mobile, USA; 5 Neurological Surgery, University of Florida College of Medicine, Gainesville, USA

**Keywords:** large language models (llms), electronic health record (ehr), electronic medical record (emr), patient care, efficiency, note writing, artificial intelligence

## Abstract

Electronic health record (EHR) systems have developed over time in parallel with general advancements in mainstream technology. As artificially intelligent (AI) systems rapidly impact multiple societal sectors, it has become apparent that medicine is not immune from the influences of this powerful technology. Particularly appealing is how AI may aid in improving healthcare efficiency with note-writing automation. This literature review explores the current state of EHR technologies in healthcare, specifically focusing on possibilities for addressing EHR challenges through the automation of dictation and note-writing processes with AI integration. This review offers a broad understanding of existing capabilities and potential advancements, emphasizing innovations such as voice-to-text dictation, wearable devices, and AI-assisted procedure note dictation. The primary objective is to provide researchers with valuable insights, enabling them to generate new technologies and advancements within the healthcare landscape. By exploring the benefits, challenges, and future of AI integration, this review encourages the development of innovative solutions, with the goal of enhancing patient care and healthcare delivery efficiency.

## Introduction and background

This review focuses on the integration of automated scribing and note dictation within electronic health records (EHRs) for clinicians in diverse healthcare settings. The objective is to explore the potential impact of automated dictation and note-writing on healthcare quality to benefit patients, providers, and all stakeholders involved. Digital scribes, known as intelligent documentation support systems, have the potential to automate the labor-intensive task of clinical documentation. Harnessing advancements in speech recognition, natural language processing (NLP), and artificial intelligence (AI) applications in healthcare, there is an opportunity to revolutionize the existing inefficient EHR systems.

Despite the growing efficiency of documentation templates, residents, physician assistants, nurse practitioners, and other healthcare professionals still spend substantial time on documentation tasks [1-7], especially when dealing with critically ill patients in settings like intensive care units (ICUs) [8,9]. The demands on modern resident physicians are multifaceted, encompassing surgical technique, decision-making, floor medicine, and extensive note-writing and administrative responsibilities.

**Figure 1:**
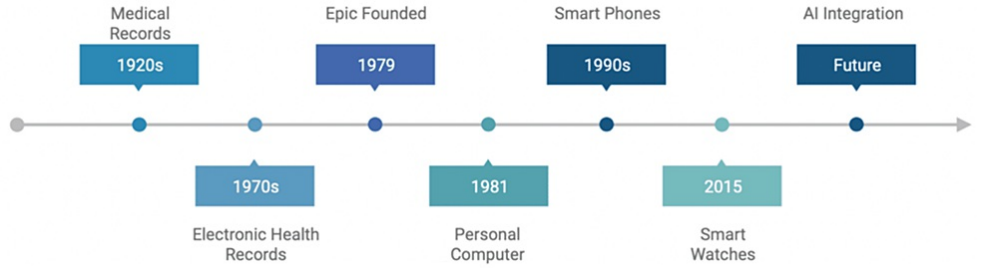
The evolution of mechanisms utilized to store and transmit health information alongside mainstream technological advances that have shaped modern society. AI: Artificial intelligence. Illustration created by Garrett Dyess.

The landscape of EHRs has undergone a transformative journey, shaped by pivotal advancements in healthcare and mainstream technology. Originating from a legacy of paper-based medical records in the early 20th century, the transition to electronic formats gained momentum in the 1960s and 70s, coinciding with the emergence of mainframe computers [10,11]. In the late 1960s, Gordon Moore, co-founder of Intel Corporation, made a groundbreaking prediction known as Moore's Law, which anticipated that the number of transistors on a microchip would double approximately every two years, leading to a significant increase in computing power and complexity [12]. During this period, Moore's foresight into the exponential growth of computing complexity became a reality, fueling the evolution of EHRs. Moore's prediction laid the foundation for the integration of advanced features such as pull-down menus, pop-up lists, and audit trails as personal computers became prevalent in healthcare settings [12,13]. This evolution, depicted in Figure 1, emphasizes the parallelism of modern technological advances alongside healthcare record-keeping systems.

In the past three decades, EHRs have undergone a remarkable transformation, evolving from physician workstations to versatile internet-based platforms accessible across a multitude of mobile devices [14,15]. Within this landscape, a select group of companies, including industry giants like Epic, Oracle, and Meditech, have dominated the market. Notably, Epic and Oracle have been at the forefront of innovation, incorporating AI technologies into EHR systems [16-18]. These AI-driven enhancements aim to extract pertinent patient information, navigate extensive datasets, and provide support to clinicians' decision-making processes [16-18]. As the healthcare paradigm shifts towards value-based care, EHR systems equipped with these capabilities are highly prioritized due to their efficiency. This strategic focus on outcomes and collaborative care is exemplified by significant industry moves, such as Oracle's acquisition of Cerner [19].

## Review

Current electronic health record challenges

The EHR has undoubtedly enhanced workflow, communication, patient safety, and compliance [20-24]. However, like many other technologies, as the EHR has advanced, it has become increasingly complex and has lost sight of its main purpose: to reduce the clerical burden on physicians [25]. Rather, physicians perceive the EHR as a clutter of data used for billing purposes, overshadowing the focus on clinical care delivery [26].

As compared to paper charting, it has been reported that resident and attending physicians may spend up to three times longer charting with the EHR [27,28]. Additionally, providers who use an EHR spend significantly less time with patients than those who use paper-based documentation [1]. This raises concern regarding how further EHR integration may continue to decrease patient-centered clinical care [1].

Multiple studies among various medical disciplines have illustrated the significant amount of time physicians spend using the EHR [2-7]. For example, a retrospective cohort study including 142 primary care physicians found that 5.9 hours of the approximately 11.4-hour workdays were spent on EHR documentation and related tasks [2]. A systematic review found that more than one-third of a physician's workday, on average, was spent on EHR tasks across various specialties [3]. Additionally, a study of general medicine residents found that 52.1% of their time was spent on EHR documentation, primarily writing notes [4]. These findings parallel those of general surgery residents at Duke University Health System, where residents were found to spend about 2.4 hours daily and 23.7 hours weekly working within the EHR [5]. Implementation of EHRs for otolaryngology residents has also been reported to result in unchanged efficiency and a decrease in direct patient care [6]. Convincingly, one study reported that general surgery residents dedicated approximately eight months to EHR usage during their entire training program [7], a significant amount of time that could be dedicated to learning, or refining, the skill sets necessary for surgical competence.

Although the widespread adoption of EHR systems across the United States (U.S.) has placed significant burdens on physicians among various disciplines, the effects on patients and the clinician-patient relationship are still debated [29-32]. Prior to the widespread implementation of EHR systems in the U.S., it was thought that a computer's presence may impede patient-physician relationships due to external interference with communication [33]. Some studies have shown no significant decline in a patient’s experience and ability to connect with their provider based solely upon computer use [29]. On the other hand, some investigations have found a negative correlation between the amount of time a provider spends using the EHR each day and surrogate markers of patient satisfaction, suggesting that the EHR may have negative impacts on patient satisfaction [30]. Given these uncertainties, it is reasonable to advise that further studies investigating patient perception, satisfaction, and computer interference be conducted to better understand such relationships.

The role of physicians has shifted from solely providing care to also encompassing extensive data-entry tasks, a potential result of increasingly complex EHR systems, which has led to a growing concern about the future of care and provider dissatisfaction with this new reality [34]. These concerns, accompanied by the advancements in AI technology, have sparked important discussions regarding the potential role of AI-EHR integration in improving the practice of medicine and reducing the time healthcare providers spend on low-yield data entry tasks [35,36].

While many systems have developed software to streamline workflow and efficiency for their specific EHR model, the standardization and interoperability between systems have not seen the same attention [37-40]. There are a number of potential reasons for this: the computational expertise and software development ability to implement, maintain, and improve a theoretical universal EHR is extensive [37,38,41,42], the potential for large-scale security breaches [42,43], and the upfront and maintenance costs of such an integrated system [37,38,44], among others. Even with advancements in technology and software development, it seems that a universal interoperability hub for all clinical applications is unattainable in the modern climate [45].

Another substantial limitation is the ethical dilemmas that present with universal synchronization. First, some entities must take responsibility for managing such a vast database. Concerns of illegal monopolization arise when that responsibility is assigned to an independent company, and significant legislative hurdles must be addressed before that responsibility can be handled by the state or federal government [46]. The combination of ethical, legislative, security, technological, and financial dilemmas of cross-platform synchronization has significantly diminished its legitimacy as a realistic solution to improved EHR access.

Historical solutions 

Although the advent of the EHR has improved certain aspects of patient care [47], the added burden on physicians has caused increased rates of burnout and dissatisfaction among providers [25,48-50]. This trend among physicians has prompted the exploration of potential solutions to help alleviate the EHR burden. In this section, previous modalities used to alleviate this burden on physicians are explored and compared.

Medical scribes are one such possibility to help lessen the EHR burden. While there is some variability depending on the institution, scribes are generally responsible for real-time documentation of the patient encounter, transcribing various lab and imaging results, and preparing instructions for discharge [51]. A randomized control trial on scribe utilization over one year revealed an increase in provider and patient satisfaction, and an increase in documentation efficiency per visit [52]. In addition to reducing clerical burdens on providers, a small observational study at one family medicine clinic showed that the use of medical scribes led to an increase in the overall joy of practice, thereby decreasing burnout among physicians [53].

While the use of medical scribes can offer undeniable benefits, there are significant drawbacks that hinder their efficacy, most notably the cost. One study on the implementation of a medical scribe program estimated the mean implementation cost to be $47,594 over the first year [54]. The main counterargument to this initial cost is the increase in productivity and patient visits that potentially would result in an overall profit for institutions. However, even a modest increase in patient volume might not be a realistic possibility for many practices [54]. In addition to the financial concerns, other drawbacks of medical scribes include the variability in the level of training, turnover rates, and potential patient uneasiness about another individual being present during medical visits [51,54]. Although the use of medical scribes has notable benefits, the significant drawbacks and an overall lack of large-study evidence limit their feasibility as a long-term solution for relieving the EHR burden on physicians.

Another avenue that many institutions have taken is the hiring of advanced practice providers (APPs) such as nurse practitioners (NPs) and physician assistants (PAs). APPs are being used more commonly due to recent physician shortages and increased patient volumes [55-57]. Studies have shown that the addition of APPs to care teams has led to decreased physician workload, especially among residents [55,57-59], which has been shown to translate to more job satisfaction and higher quality work [57,60,61]. 

While the benefits of APPs are clear, there are certain drawbacks to consider. APPs undergo training in general medicine, but they do not have standardized training in areas of specialty, meaning that this training falls on a specific site [56]. While this is manageable for larger academic institutions, smaller facilities might struggle to provide adequate training or find suitable APPs willing to work there [56]. Another consideration is the financial aspect of hiring APPs. For a major teaching hospital, it costs roughly $3.2 million annually for APPs to effectively reduce the resident workload for a general medicine residency program [57-59].

Aside from decreasing workload via scribes or APPs, alterations to the EHR itself, either with unique templates or modifications to usability have also been explored [62-65]. One hospital system implemented a Computerized Provider Order Entry (CPOE) system to decrease the number of clicks for electronic ordering for its neonatal intensive care unit (NICU) faculty and found that overall provider job satisfaction increased [63]. Another institution developed an enhanced EHR designed to streamline abnormal laboratory result delivery to providers by prioritizing abnormal and critical results of patients who did not have a scheduled follow-up evaluation, and additionally offered instructions for the next steps [62]. The providers that utilized the enhanced EHR system indicated lower cognitive workload and improvement in clinical performance [62].

While many studies have shown that modifications to the EHR can improve the burden on physicians, they also highlight ­­key drawbacks. Many modifications tend to be hyper-specific to a certain specialty, provider group, or system, meaning that their effectiveness is limited to a select group [62-64,66]. In the CPOE study, for instance, the change in order delivery resulted in a disruptive nursing workflow, resulting in hesitancy and lack of comfort among the nursing staff [63]. Furthermore, a randomized control trial conducted to compare a new, succinct note template to a standard template showed that while shorter and less redundant, the new template was less organized and was not of any higher quality [65]. This further challenges the issue of constructing an effective, efficient template. It is difficult to implement a modification into the EHR that is universally beneficial for all parties, and further studies are needed to elucidate the practicality of large-scale EHR modifications and their effectiveness in decreasing the EHR burden.

Though solutions to the EHR burden have been offered over time, there is still much left to be desired in achieving a long-term fix. The benefits and hindrances of the commonly utilized implementations to reduce the clerical burden on physicians are summarized in Table 1.

**Table 1:** Summary of benefits and drawbacks of commonly utilized solutions to alleviate the EHR burden. APP: Advanced practice provider; EHR: Electronic health record.

Solution	Attributes		
	Description	Benefits	Drawbacks
Medical scribes	Real-time documentation, transcribing results, and discharge instructions.	Increased provider and patient satisfaction and improved documentation efficiency [52].	High initial cost [54], variable training levels, patient uneasiness, and increased turnover rates [51,54].
APPs	Nurse practitioners and physician assistants.	Reduced physician workload [55,57-59], increased job satisfaction, and higher quality work [57,60,61].	Lack of standardized training [56], and financial costs for training and hiring [57-59].
EHR modifications	Unique templates, usability enhancements, and streamlined order entry.	Improved provider satisfaction [63], decreased cognitive workload, and streamlined workflows [62].	Specialty-specific, disruptive workflows, lack of universal effectiveness, and need for further studies [62-66].

Benefits of automated note writing in clinical practice

Automated dictation and note-writing can have numerous benefits in clinical practice. Primary benefits include the ability to streamline real-time workflow, increase patient satisfaction, and improve note accuracy. Despite the convenience and necessity of EHRs, it has become increasingly apparent that clinicians dedicate a large portion of their time to documentation [1-7,67,68].

Automation in the form of digital scribe systems can potentially mitigate this issue. Digital scribe systems utilize emerging technologies, such as speech recognition and NLP, to automatically convert a verbal patient encounter directly into a summarized, well-structured medical chart [69]. These systems, in theory, would allow physicians to fully dedicate their complete attention to their patients without becoming routinely distracted throughout the encounter by entering their notes via keyboard and mouse [70]. Patients notice these distractions, often citing the uncomfortably long pauses while their physicians type their notes as a fundamental source of dissatisfaction during an encounter [30]. Instead of splitting their time interfacing with a computer, clinicians could dedicate their undivided attention to actively speaking with their patients, thus improving one-on-one patient interaction. These changes can potentially have a marked positive effect on the quality of health outcomes for patients. Several studies have determined that improved health outcomes, such as medication compliance, reduction in negative emotions, and overall improved general well-being, are correlated with changes in physician behavior during encounters [71,72]. These studies report that deep conversations where clinicians demonstrate empathy for a patient’s experience or nonverbal behaviors, such as physical touch or eye contact, can bring about these improvements in health outcomes and improve the trust relationship between provider and patient [72-76]. With the assistance of digital scribe systems, physicians can dedicate more of their attention to these important behaviors, thus improving their overall care.

In addition to enhancing one-on-one patient interactions, automated dictation, and note-writing can assist providers with improving their clinical accuracy. Automation can achieve this goal via various modalities. For example, recent efforts have been made to determine if automation can assist in resident and fellowship training, particularly those in procedural specialties [77]. This study developed a structured template that will automatically create a procedural log for interventional cardiology procedures using the documented clinical record [77]. It was determined that this system, compared to manual entries completed by fellows, contained more detail per case while reporting more procedures, thus highlighting its practical efficiency, as well as its potential ability to validate and teach fellows [77]. While these findings are important to consider, these highly specific templates come with considerable drawbacks, as mentioned previously.

Furthermore, automation can play a potential role in medical billing. Recent studies demonstrate that incorrect billing code use, primarily via international classification of disease 10 (ICD-10) coding, is an ongoing issue faced in hospitals [78]. Automation, powered by natural language processing-bidirectional recurrent neural network (NLP-BIRNN) algorithms, can provide a useful tool for utilizing existing clinical records in EHRs to verify and correct ICD-10 coding conducted by human personnel [78]. Another study investigated the performance of convolutional neural networks in predicting ICD-10 codes using existing clinical notes and reported clinically satisfactory performance [79]. Together, these studies illustrate the potential capability of automation in preventing medical errors in patient charts, thus reducing medical waste, limiting administrative burden, and improving overall patient care.

Automation possibilities and future directions

Voice-to-Text

Although automated dictation and note-writing can offer many benefits to clinicians, it is important to consider potential limitations and drawbacks. A challenge with automation in medicine is the lack of transparency in decision-making. This makes it difficult for clinicians to understand how the system arrived at a particular diagnosis or organization of patient notes. Furthermore, there are limits to the scope of AI-based systems, which can lead to unexpected errors or inaccuracies in patient records. These issues highlight the need for physicians to use AI systems as decision-support tools rather than definitive diagnostic tools. Despite these limitations, voice-to-text dictation has gained traction in the healthcare industry, but limited evidence exists on its effect on physician efficiency and patient outcomes [10]. The utilization of voice dictation systems also varies among healthcare professionals with different dictation needs depending on their role in patient care. 

While existing studies suggest a trend towards improvements in turnaround time for patients and cost-effectiveness with the use of voice dictation systems [11,13,16], significant questions regarding patient outcomes, dictation accuracy, and resource allocation have not been fully elucidated. It is still necessary to determine which healthcare workers are most likely to benefit from voice-to-text dictation. One area of development that could help address these challenges is NLP, a computational process that enables machines to understand and generate human language. Advances in NLP may increase the accuracy and practicality of implementing text-to-voice dictation systems across a wider audience of healthcare professionals.

Prior attempts to increase note efficiency include the introduction of note pre-made templates. Research has shown that the use of pre-made templates can potentially result in better outcomes, particularly in work tasks with high overlap and rapid pace of output [16]. The combination of improved scribing through advancements in NLP and intelligent systems that can accurately place encoded information into the correct templates for specific work tasks represents a promising direction for future research in this field. This may lead to enhanced benefits of voice-to-text dictation while minimizing common errors.

Procedure Note Dictation

One way that AI integration could transform procedural-based medicine is the possibility of leveraging audio and video capture technology to record and document data in real-time, directly into the patient's health record [80,81]. While it has yet to be extensively studied, AI-assisted procedure note dictation may decrease the amount of time spent on manual documentation while refocusing residents' attention on mastering their skill sets and pursuing other avenues of career training [7]. This may also revolutionize the way physicians capture and store patient information, ultimately leading to improved accuracy, efficiency, and patient outcomes [81,82]. However, the attainability of such an integration comes with a significant set of challenges, including ethical and legal concerns for both patients and physicians alike.

Currently, AI advancements in the specific fields of computer vision and NLP are increasing the capability of computers to interpret visual input, including static, kinematic, and dynamic motion, and the ability to interpret and act on text and voice data [80,82,83]. AI-mediated visual and audio data capture and interpretation may be an effective starting point to increase the efficiency of operating room logistics, including documentation. Currently, there are AI-integration software platforms that can capture clinician audio input, interpret, and document within the EHR, albeit not widely used due to significant limitations with accuracy and clinician hesitancy [82,84,85]. There is significant interest in creating new AI-NLPs that can automatically interpret and document clinical encounters between physicians and patients [84]. This is a plausible option for multiple different medical specialties; however, this would certainly require more intricate AI capabilities than are currently available.

Wearable Monitoring Systems

The use of innovative technologies such as wearable devices has emerged as a promising area of research, with significant advancements being made in both outpatient and inpatient settings. The field of wearable devices for medical purposes is undergoing rapid advancements, encompassing real-time monitoring of vital signs in intensive care units, and the development of mobile electroencephalogram (EEG) and electrocardiogram (EKG) monitors for outpatient care, among others [17,18,86-88].

While certain wearable devices, such as the Apple Watch (Apple Inc., Cupertino, California), have gained widespread popularity, others are still relatively unknown. In medical contexts, particularly in intensive care units (ICUs), the utilization of medical sensors in wearables for real-time monitoring of vital signs is crucial for optimizing patient care. Furthermore, wearables designed for outpatient care, such as mobile EEG and EKG monitors, are also being actively developed [17,18].

Notably, the pivotal role of algorithms cannot be overstated in harnessing the potential of data generated by wearable devices. While wearables in outpatient settings are poised to improve with ongoing technological innovation, the sheer volume of data to be analyzed poses a significant challenge. AI-enhanced wearable devices may soon be able to provide accurate interpretations of EEGs and EKGs, a feat that would help limit the volume burden on physicians who are currently tasked with these interpretations [89,90].

Wearables used in conjunction with cellular devices could also be used to document subjective patient health data such as pain descriptors, symptom specifiers, and psychological factors. Having an electronic recording device nearby routinely provides an excellent opportunity to collect and capitalize on previously non-relayed information, albeit increasing the demand for systems that can accurately analyze and properly report important health concerns.

Major limitations

In this section, the challenges hindering the integration of AI in healthcare are explored, considering various modalities of AI-based automation and their respective implications. Clinician hesitancy arises due to inconsistencies and biases in automated clinical decision support algorithms (ACDSAs), impacting trust and clinician vigilance. Ethical concerns surface with audio and video technologies, necessitating patient consent and stringent data security protocols. Legal issues encompass data tampering risks and compliance complexities, adding to apprehensions. Synchronization challenges in EHRs and the 'black-box phenomenon' in AI decision-making further complicate seamless integration.

Clinician Hesitancy

ACDSAs have become a particularly interesting tool for diagnosis and treatment. Studies have shown that the use of ACDSAs results in improvements in the interpretation of clinical data, evaluating prognostic factors for illness, improving productivity, and reducing medical errors [91-94]. However, while these systems have a potential role in limiting clinical errors, they also contribute some of their own. For example, image interpretation algorithms have been shown to be inconsistent when presented with images from outside of a standard training set, and even minor alterations to an image can drastically alter the algorithm's performance [95-98]. 

Additionally, algorithms can incorporate the values and biases of their data writers. For instance, algorithms can prioritize minimization of false negatives over false positives or vice versa, or perform differently for differing socioeconomic groups; after all, an algorithm is evolved from mass amounts of data introduced to it by its developers to shape its abilities [99,100]. Shortcomings of ACDSAs increase the hesitancy to trust these systems among clinicians [101], which is likely unamenable until consistent reliability can be achieved in a clinical setting. This presents another issue to consider: variable levels of confidence in algorithms. While reliability for an algorithm is obviously a necessity, there must be an appropriate level of trust given by a clinician to an algorithm [102,103].

In a perfect scenario, the clinician’s medical decision is not made by the algorithm, but rather reinforced by it [94,104]. Excessive dependence on an automated system can result in decreased clinician vigilance and increased risk of clinical catastrophe. Moreover, as more reliance is placed on an algorithm to assist in clinical decision-making, less weight is given to the sharing of thought processes and reasoning between clinician colleagues [105]. On the contrary, insufficient trust in an algorithm can result in decreased efficiency and delayed clinical decision-making [102,103]. Attaining a balance of reliance and trust in algorithms is essential for their effectiveness, although this will not be easy.

Ethical Concerns of Audio and Video Technologies

Although it is evident that the future of AI assistance in the clinical setting may be extraordinarily beneficial, it comes with challenges beyond its technical constraints. While basic audio and video capture technologies have been used for educational purposes in the past [106,107], they may pose more significant ethical challenges if they were to become more widespread and incorporate AI without the sole intention of enhancing medical education. The most important stakeholder of this ethical concern is clearly the patient. In the case of any medical intervention, a patient's autonomy is of the utmost importance. Prior to being recorded, patients would need to provide informed consent to be recorded, understand why they were being recorded, know of the individuals that may have access, and know when or how the recorded data would be stored or destroyed [81,108]. Such an integration would inherently increase the demand for increased data storage capacity, encryption, and destruction processes to ensure patient privacy and confidentiality are adequately maintained [109]. Lastly, access to large amounts of clinical video and audio data would be necessary to train an AI system to be able to complete these tasks [82,110]. This need for extensive data poses further ethical concerns regarding protected health information and access to such data, making the development of such a system a challenging task for researchers and software developers [110].

Legal Concerns of Audio and Video Technologies

Legal implications play a significant role in the discussion for audio and video recordings in general and for AI-integration applications as well [81,109,111]. Physicians already express concern about using video recording devices when it could be clinically beneficial due to fears of violating the Health Insurance Portability and Accountability Act and due to the videos being harmful regarding malpractice suits [109,112]. In the case a malpractice suit is filed against a physician, it may be possible for a court to obtain access to such data to help establish the facts of a case when negligence is suspected [108]. Although a positive outcome in the case of true negligence to a patient may prove unfair, in the instance that it cannot provide a complete context of what may have occurred, allowing a potential false interpretation of misconduct. Even though these concerns are valid, in situations where videos are used in malpractice suits, most of them support the case of the physician [109,113]. Additionally, these recordings may be subject to tampering by either party if not properly protected, allowing for misrepresentation of a clinical scenario. One potential solution to lessen this legal concern could be to integrate an inherent AI-generated de-identification process via video processing techniques, as evidenced was possible in a study in 2015 conducted by Silas et al. [111]. Legal challenges presented here clearly indicate the need for comprehensive and rigorous guidelines and procedures for addressing these concerns before incorporating AI-integrated video and audio recording technologies into the clinical space.

Transparency and Extrapolation

One significant limitation of implementing AI-based automation in medicine is the lack of transparency in decision-making, termed the ‘black-box phenomenon.’ The algorithms supporting AI can often be opaque and difficult to understand, thus creating a challenge for clinicians to understand how the system arrived at a particular diagnosis, billing code, or organization of patient notes [114,115]. Additionally, a major limitation of AI-based systems is their limit in scope. AI can only operate within the predefined parameters it has been trained on. Thus, extrapolating these systems to different specialty clinics or facility types (outpatient, inpatient, operating room, etc.) can create unexpected errors or inaccuracies in patient records, all of which can lead to the endangerment of patients and medical resource waste [116]. The lack of evidence to support the diverse accuracy of these models in various healthcare environments promotes a pervasive attitude about AI systems in clinical practice: physicians must use them as decision-support tools instead of definitive diagnostic tools [114]. Although AI systems have powerful applications, it will likely remain the duty of the clinician to make finalized medical diagnoses and treatment plans for their patients.

Collectively, the complexities discussed bring to light the importance of cautious implementation, balancing innovation with ethical, legal, and practical considerations, and essentially shaping the trajectory of AI's effective utilization in healthcare. These challenges and potential consequences that must be considered are summarized in Table 2.

**Table 2:** Summary of challenges and possible consequences of general AI automation in healthcare. AI: Artificial intelligence; EHR: Electronic health record.

AI challenge	Possible consequence
Decreased clinician vigilance	Over-reliance on AI systems may lead to decreased attention to critical clinical details [101-103].
Risk of clinical errors	Inconsistencies and biases in AI algorithms can lead to clinical errors and compromised patient safety [99,100].
Patient privacy concerns	Patient consent, data storage, and access to recorded audio and video data raise ethical and privacy issues [81,108,109].
Fear of malpractice suits	Clinicians fear potential legal implications and malpractice suits due to recorded data [109,113].
Inefficiencies in EHR access	Lack of synchronization and interoperability results in inefficiencies in accessing patient records [116].
Monopolization concerns	Ethical and legal dilemmas become apparent regarding the management and control of a universal EHR database [46,116].
Errors in patient records	Lack of transparency and limited scope in AI systems can result in errors in patient records [114,115].

## Conclusions

The integration of artificial intelligence (AI) technologies into the medical charting system marks a crucial milestone in healthcare advancement. Despite significant progress in the past three decades, the burden on healthcare professionals persists. AI-driven solutions, such as voice-to-text dictation and automated procedure note dictation, offer expansive potential. Voice-to-text dictation, enhanced by natural language processing, not only improves accuracy but also streamlines workflow, enabling clinicians to prioritize direct patient care. These innovations address inherent flaws in the current electronic health record system, providing an opportunity to mitigate existing issues.

However, the introduction of AI in healthcare necessitates careful planning and rigorous testing due to the need for safety and protection. Ethical and legal challenges are important considerations as protecting patient privacy and data security is of the utmost importance. Wearable monitoring systems, coupled with AI, may deliver real-time patient data, facilitating quicker diagnosis and treatment. While challenges like cross-platform synchronization complexities and trust in automated clinical decision support algorithms persist, a balanced ethical approach is essential. AI-enhanced healthcare promises reduced clinician burdens and significant healthcare advancement, however, embracing a holistic and ethical approach will be crucial for maximizing AI's utility in modern medicine.
